# Supplementary material for: Patterns of Intron Gain and Loss in Fungi
Source: PLoS Biol. 2004 Nov 30;2(12):e422. doi: 10.1371/journal.pbio.0020422 (PMC532390; doi:10.1371/journal.pbio.0020422)
Supplement: Table S1 — Also available at http://genes.mit.edu/NielsenEtAl/. (4.3 MB ZIP). [file pbio.0020422.st001.zip › NielsenEtAl/html/1328.html]

AN4998.1.NCU06122.1.MG03846.1.FG00355.1


```
 CLUSTAL W (1.82) Multiple Sequence Alignments - Introns Inserted


Sequence 1: NCU06122.1	770 aa
Sequence 2: MG03846.1	752 aa
Sequence 3: FG00355.1	752 aa
Sequence 4: AN4998.1	751 aa
Alignment Length: 770 aa
Number Identitical Residues: 557 aa
Alignment Score (without introns) 23291


MG03846.1 	MSSYDGSRSTRQSKRYSMSALYMSMSANETDLEIEDDLAKA1QKVLRDLKSKISSQSKKN
NCU06122.1	MSSYEGSRSTRQSKRYSMSALYMSMSANDSDLIIEDELARA1QKVLRDLKTKISSQSKKN
FG00355.1 	MSSYDGSRSARQSKRYSMSALYMSISANEGDLQIEDELAKA1QKSLRDLKSKISSQSKKN
AN4998.1  	MSSHD-TRSLRQSKRMSVTALYLSMSAKDRDLEISDDLARA1QKFLRELKSKISSQSKKN
          	***:: :** ***** *::***:*:**:: ** *.*:**:* ** **:**:*********

MG03846.1 	FVLEKDVRYLDGRIALLIQNRMALEE0QHETASYLEDAADLQEGAFPNDDRTQKYGNLMF
NCU06122.1	FVLEKDVRYLDSRIALLIQNRMALEE0QNEVASHLEDATDIQEGAFPNDDKTQKYGNLLF
FG00355.1 	FVLEKDVRYLDSRIALLIQNRMALEE0QNEVASHLEDALEMQQGGFPNDDKTQKYGNLLF
AN4998.1  	FVLEKDVRYLDSRIALLIQNRMALEE0QNEVANRLDDTVDPQEGFFPNDEKTQKYGNLLF
          	***********.************** *:*.*. *:*: : *:* ****::*******:*

MG03846.1 	LLQSEPRHIAHLCRLVSMQEIDSLLQTVMFTIYGNQYESREEHLLLTMFQ0SVLTYNFEN
NCU06122.1	LLQSEPRHIAHLCRLVTMAEIDSLLQTVMFTIYGNQYESREEHLLLTMFQ0SVLTYQFDN
FG00355.1 	LLQSEPRHIAHLCRLVSMSEIDSLLQTVMFTIYGNQYESREEHLLLTMFQ0SVLTYQFDN
AN4998.1  	LLQTEPRHIAHLCRLVSMSEIDSLLQTVMFTIYGNQYESREEHLLLTMFQ~SVLTYQFDN
          	***:************:* ******************************* *****:*:*

MG03846.1 	TPEYSSLLRANTPVSRMMTTYTRRGPGQSFLKSVLADKINSLIELRDLDLEINPLKVYER
NCU06122.1	TPEYSSLLRANTPVSRMMTTYTRRGPGQSFLKSVLADRINSLIELKDLDLEINPLKVYER
FG00355.1 	TPDYSSLLRANTPVSRMMTTYTRRGPGQSFLKSVLADRINGLIELKDLDLEINPLKVYER
AN4998.1  	TPEYSSLLRQNTPVSRMMTTYTRRGPGQSYLKQVLAEQINALIELRDVDLEINPLKVYET
          	**:****** *******************:**.***::**.****:*:*********** 

MG03846.1 	MIEQIEEDTGSVP-PSLPRGVTAEQAAANPQVQEIIEPRLAMLTDIANGFLSTIIDGLEE
NCU06122.1	MIEEAEAKDPESPEAMLPKGITQEEAAENPKVQEIINPRLTMLTEIANGFLETIIDGLEE
FG00355.1 	MIEQIEEDTGQLP-PHLPKGITGEQAAENPQVQAIIEPRLTMLTEIANGFLTTIIEGLEE
AN4998.1  	MVRDIEEETGSLP-DHLPRGVTGEVAAENPQVQAIIAPRLKKLTEIANSFLTTIINSVNQ
          	*:.: * .  . *   **:*:* * ** **:** ** ***  **:***.** ***:.:::

MG03846.1 	APYGIRWICKQIRSLTKRKYPDANDQVICTLIGGFFFLRFINPAIVTPKSYMLIDGQPAD
NCU06122.1	TPYGIRWICKQIRSLTKRKYPDANDQIICTLIGSFFFLRFINPAIVTPKSYMLIDGTPAD
FG00355.1 	APYGIRWICKQIRSLTKRKYPDANDQVICTLIGGFFFLRFINPAIVTPKSYMLIDGTPAE
AN4998.1  	APYGIRWICKQIRSLSRRKYPDAHDQTICTLIGGFFFLRFINPAIVTPRSYMLIDATPTD
          	:**************::******:** ******.**************:******. *::

MG03846.1 	KPRRTLTLIAKMLQNLANKPSYAKEPYMAGLQPFVQMNKSRVNKFMLDLCEVGDFYESLE
NCU06122.1	KPRKTLTYVAKMLQNLANKPSYAKEPYMAKLQPFIHANKDKVNRFMLELCEVQDFYETLE
FG00355.1 	RPRRTLTYIAKMLQNLANKPSYAKEPYMAKLQPFIHQNKDRINKFMIDLCEVQDFYESLE
AN4998.1  	KPRRTLTLIAKMLQNLANKPSYAKEPYMAKLQPFIQQNKERVNKFMLDLCEVQDFYESLE
          	:**:*** :******************** ****:: **.::*:**::**** ****:**

MG03846.1 	MDNYVALSKKDLELSITLNEIYAMHGLLEKHAHELCRDENSHLALLMGELGQAPQQVPRK
NCU06122.1	MDNYVALSKKDLQLDITLNEIYAMHGLLEKHAGELYKDENSHLAIIMSELGTAPPQVPRK
FG00355.1 	MDNYVALSKKDLELEITLNEVYAMHSLIDKHHSELWKDDNSHLAIIMSELGSSPPQLPRK
AN4998.1  	MDNYVALSKRDLELQITLNEMYATHALLEKHNAALAQDQHSHLQEILQELGPAPPQLPRK
          	*********:**:*.*****:** *.*::**   * :*::***  :: *** :* *:***

MG03846.1 	ENRVMNLPLYSRWETAIDNLTAALDITQEEVFFMEAKSIFVQIMRSIPQNTAVARRPLRL
NCU06122.1	ENRVINLPLFSRWETAIDDLTAALDITQEEVFFMEAKSIFVQILRTIPANSSVARRPLRL
FG00355.1 	ENRVINLPLFSRWESAIGDLTAALDITQEEVYFMEAKSIFVQVMRSIPSTSGVARRPLRL
AN4998.1  	ENRTITVPLFSRWETALDDLTSALDITQEEVFFMEAKSTFVQILRSLPPHSSVARRPLRL
          	***.:.:**:****:*:.:**:*********:****** ***::*::*  :.********

MG03846.1 	ERIADAAATSKNDAVMVRKGIRAMELLSQLQELKVTDKSDGFGLLRDEIEQELQHLGSLK
NCU06122.1	ERIADAAATSKNDAVMVRKGIRAMELLSQLQELKVVDKSDQFSLLRDEVEQELQHLGSLK
FG00355.1 	ERIADAAATNRSDAVMVRKGIRAMELLSQLQEMHVIDKTDQFSLLRDEVEQELQHLGSLK
AN4998.1  	DRIAEAAATLKNDAVMVRKGIRTMELLSQLQEMGVIDRSDEFSLLRDEVEQELVHLGSLK
          	:***:**** :.**********:*********: * *::* *.*****:**** ******

MG03846.1 	EGVIAETQKLEEVYKTIRDHNAYLVGQLETYKSYLHNVRSQSEGTRRKAQKQQVLGPYKF
NCU06122.1	EVVMSETIKLEEVYKTIRDHNAYLVSQLDTYKSYLHNVRSQSEGKTRKQQKQQVLGPYKF
FG00355.1 	EGVIAETSKLQEVYKTIRDHNVYLNGQLETYKSYLHNVRSQSEGTKRKQQKQQVLGPYKF
AN4998.1  	EKVMEETRQLESVYATIRDHNAYLVGQLETYKSYLQNVRSQSEGKSRKTQKQQELGPYKF
          	* *: ** :*:.** ******.** .**:******:********. ** **** ******

MG03846.1 	THQQLEKEGVIQKSNVPDNRRANIYFNFTSPLPGTFVISLHYKG1---------------
NCU06122.1	THQQLEKEGVIQKSNVPDNRRANIYFNFTSPLPGTFVISLHYKG~ESPELPSTMSNTDSS
FG00355.1 	THQQLEKEGVIQKSNVPDNRRANIYFNFTSPLPGTFVISLHYKG1---------------
AN4998.1  	THQQLEKEGVIHKSNVPENRRANIYFMFKSPLPGTFVISLHYKG~---------------
          	***********:*****:******** *.***************                

MG03846.1 	--RTRGLLELDLKLDDLLEMQKDGQDDLDLEYVQFNVTKVLALLNKRFARKKGW
NCU06122.1	VGRNRGLLELDLKLDDLLEMQKDNQDELDLEYVQFNVPKVLALLNKRFARKKGW
FG00355.1 	--RNRGLLELDLKLDDLLEMQKDNQDDLDLEYVQFNVPKVLALLNKRFARKKGW
AN4998.1  	--RARGLLELDLKLDDLLEMQKDNLEDLDLEYVQFNVSKVLTLLNKRFSRKKGW
          	  * *******************. ::**********.***:******:*****
```
